# Incidental Detection of Tracheobronchopathia Osteochondroplastica During a Bronchoscopy in a Patient With Papillary Thyroid Cancer: A Case Report

**DOI:** 10.7759/cureus.83776

**Published:** 2025-05-09

**Authors:** Tarek Zaki, Moustafa S Mashal, Mohammed AlAttar, Alshimaa Almashad, Julio Gómez-Seco

**Affiliations:** 1 College of Medicine, University of Sharjah, Sharjah, ARE; 2 Faculty of Medicine, Cairo University, Cairo, EGY; 3 Internal Medicine Department, Fakeeh University Hospital, Dubai, ARE; 4 Pulmonology Department, Fakeeh University Hospital, Dubai, ARE

**Keywords:** airway obstruction, bronchoscopy, chronic infection, nodules, trachea, tracheobronchopathia osteochondroplastica

## Abstract

Tracheobronchopathia osteochondroplastica (TPO) is a rare, benign condition characterized by submucosal osseocartilaginous nodules sparing the posterior tracheal wall. We report a unique case of a 45-year-old male with a history of smoking, gastroesophageal reflux disease (GERD), and papillary thyroid carcinoma, in whom TPO was incidentally discovered during intraoperative bronchoscopy for hemithyroidectomy. The patient was asymptomatic from a respiratory standpoint, but bronchoalveolar lavage identified *Streptococcus pneumoniae* and methicillin-resistant *Staphylococcus aureus* (MRSA), suggesting a possible role of chronic infection in TPO pathogenesis. Diagnosis was confirmed through imaging and histopathology, revealing typical tracheal nodularity with no airway obstruction. No specific treatment was required for TPO, and the patient was managed conservatively with antibiotics for airway colonization. This case highlights the importance of recognizing TPO during unrelated procedures to avoid misdiagnosis and supports the hypothesis of infection-driven chronic inflammation contributing to its development.

## Introduction

Tracheobronchopathia osteochondroplastica (TPO) is a rare and often underdiagnosed disorder, characterized by submucosal osseocartilaginous nodules in the trachea and large airways [1]. These nodules can lead to focal airway narrowing due to cartilage and bone formation in the airway wall, along with calcium phosphate deposition [2]. The disease was first described by Wilks in 1857 as ossific deposits in the airways [3], although it was initially identified at autopsy by RoRitanky in 1855 [4]. Since TPO is usually asymptomatic, the true prevalence of TPO remains unclear. Approximately 500 cases had been reported worldwide by April 2017 [5]. In this case report, TPO was incidentally discovered in the patient during a thyroidectomy procedure performed for papillary thyroid carcinoma. 

A 2020 review of literature found 60 reported cases of TPO in PubMed between 2010 and 2018, with 60% occurring in females and patient ages ranging from 20 to 80 years [5]. Other studies report that it usually presents between the ages of 25 and 85, with peak incidence in the fifth decade of life and no clear gender predominance [6]. 

The condition is underdiagnosed mainly because of its rarity, variable presentation, and low physician awareness. Its symptoms, such as chronic cough, dyspnea, hemoptysis, hoarseness, or wheezing, can resemble more common respiratory diseases [7]. Bronchoscopy is the gold standard for diagnosis, allowing direct visualization of the lesions and sampling for pathogens in recurrent infections [2]. Important differentials include amyloidosis, relapsing polychondritis, mucoepidermoid carcinoma, papillomatosis, and sarcoidosis [8]. Given its nonspecific presentation and potential for incidental discovery, TPO remains a diagnostic challenge that warrants increased clinical awareness and thorough investigation when airway abnormalities are encountered.

## Case presentation

**Figure 1:**
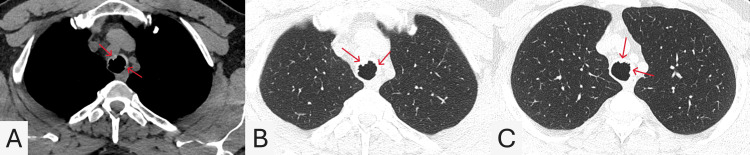
Axial non-contrast computed tomography (CT) chest images (A-C) (A) Soft tissue window shows irregular nodular thickening with calcified submucosal densities along the anterior tracheal wall (red arrows), sparing the posterior membranous wall. (B) Lung window highlights the extent of anterior wall nodularity (red arrows) without significant luminal narrowing. (C) A slightly lower lung window level shows submucosal densities (red arrows) with sparing of the posterior tracheal wall and a preserved airway lumen.

**Figure 2:**
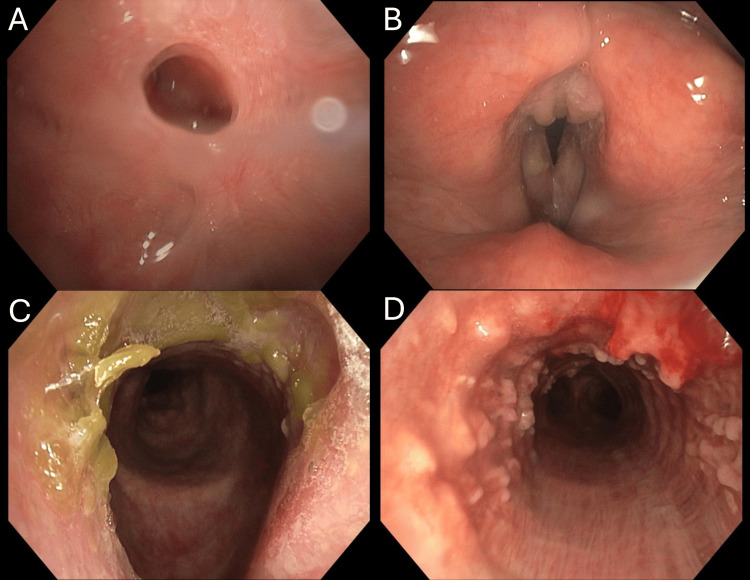
Flexible bronchoscopy images (A-D) (A) A 3-4 mm cold ulcer/perforation of the hard palate. (B) Exophytic inter-arytenoid lesions and two polypoid/leukoplakic lesions on the right vocal cord. (C) Bright green crusted secretions in the subglottic area. (D) Multiple papillomatous/exophytic nodules on the anterior and lateral walls.

A 45-year-old male, with a history of smoking and gastroesophageal reflux disease (GERD), was scheduled for a right-sided hemithyroidectomy following the diagnosis of papillary thyroid carcinoma. During the surgical procedure, the anesthesiologist performed an intraoperative bronchoscopy to assist with intubation, which revealed multiple exophytic nodular lesions on the anterior tracheal wall, raising suspicion for TPO. A subsequent non-contrast computed tomography (CT) scan of the chest demonstrated irregular nodular thickening along the anterior wall of the trachea, predominantly involving its lower third (Figure 1). The nodules appeared as small submucosal calcified densities, consistent with osseous or cartilaginous lesions. Notably, the posterior membranous wall was spared - a characteristic radiological finding of TPO - with no evidence of significant tracheal narrowing or airway obstruction. The lung parenchyma and mediastinal lymph nodes appeared unremarkable. Flexible bronchoscopy further confirmed the presence of multiple exophytic, papillomatous lesions along the anterior and lateral walls of the trachea, as shown in Figure 2D. In addition, bronchoscopy revealed a 3-4 mm cold ulcer/perforation with a central line on the hard palate, as can be seen in Figure 2A. Exophytic lesions were also observed on the inter-arytenoid membrane, along with two polypoid or leukoplakic lesions on the right vocal cord (Figure 2B). 

**Figure 3:**
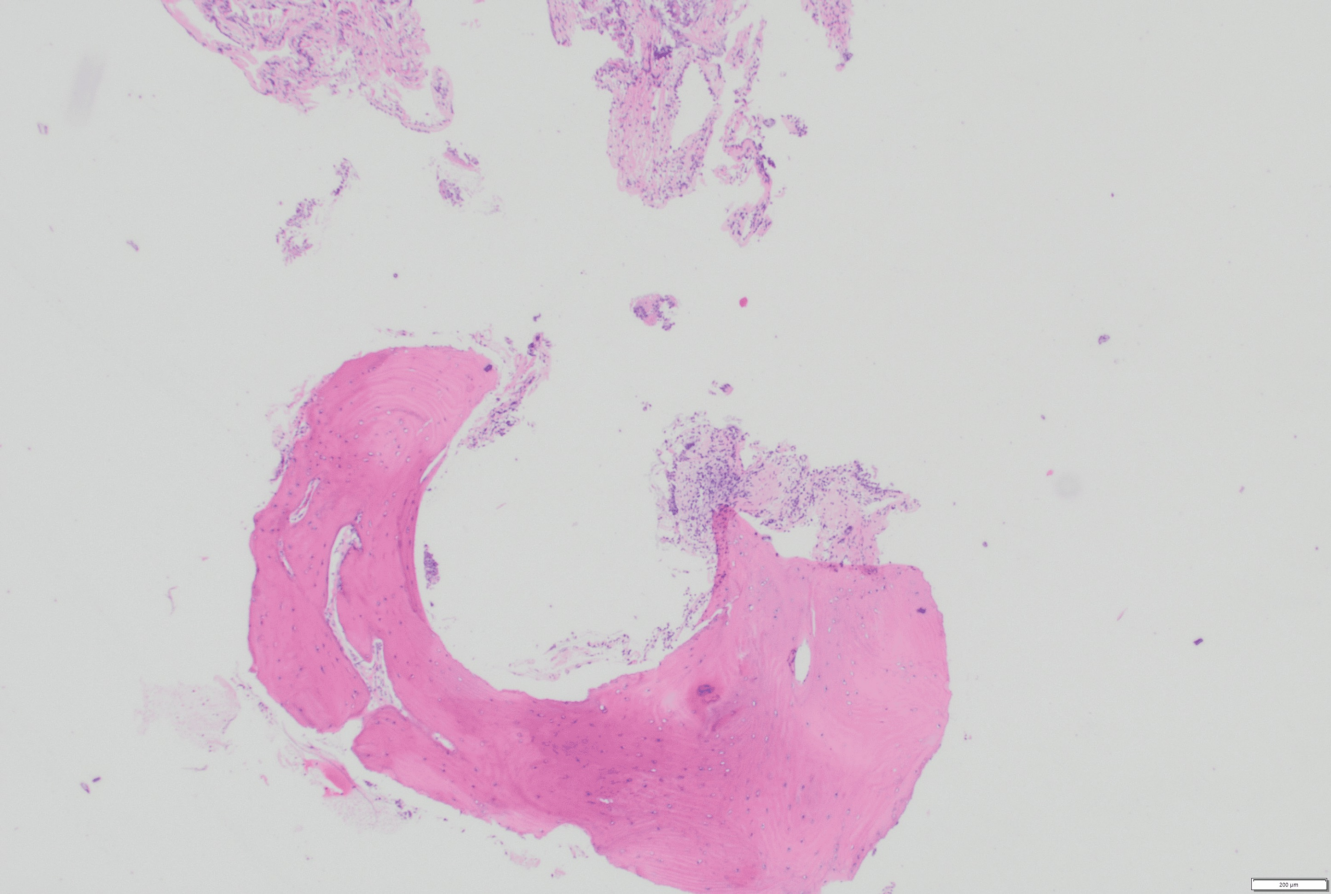
Histopathological section of a tracheal nodule Histopathology showing small fragments of bony tissue lined by the respiratory mucosa, with submucosal edema and mixed acute and chronic inflammatory infiltrates.

Histopathological examination of the biopsy specimens revealed benign small bone fragments with overlying respiratory mucosa, with no evidence of atypia or malignancy (Figure 3). Bronchoalveolar lavage (BAL) cytology showed mild chronic inflammation, and microbiological cultures identified the presence of *Streptococcus pneumoniae* and methicillin-resistant *Staphylococcus aureus* (MRSA), despite no clinical symptoms of respiratory infection. ENT referral was obtained, and the bacterial infections resolved after a course of moxifloxacin and amoxicillin-clavulanate. No specific intervention was pursued for TPO, given the benign nature of the condition and the absence of significant airway obstruction; however, follow-up with bronchoscopy and CT imaging was advised in 12 months. 

## Discussion

This case report discusses TPO, a rare, benign airway disorder characterized by submucosal osseocartilaginous nodules that spare the posterior membranous wall [1]. Although frequently asymptomatic, it may present with non-specific respiratory symptoms that often lead to misdiagnosis [7,9]. Its incidence is estimated at one in every 2,000 bronchoscopies [10]. The diagnosis of TPO is often incidental, confirmed via imaging, endoscopic visualization, or histopathological examination [11]. Bronchoscopy enables direct assessment and biopsy of lesions, while histology typically shows metaplastic cartilage and bone with chronic inflammation. Management is generally conservative in asymptomatic cases [5]. However, in patients with airway compromise, endobronchial interventions such as curettage, Argon plasma coagulation, or stenting may be necessary [11,12]. The lack of standardized treatment guidelines reflects both the rarity and typically indolent course of the disease. 

The exact etiology of TPO remains unknown, although several hypotheses exist. Proposed mechanisms include chronic infection, congenital anomalies, chemical or mechanical irritation, metabolic disturbances, chronic inflammation, and amyloidosis, of which inflammation appears to be the most plausible contributor [13,14]. In this case, the isolation of *Streptococcus pneumoniae* and MRSA raises the possibility of infection-driven pathogenesis. Our findings support the hypothesis that microbial colonization may serve as a persistent irritant, which initiates chronic inflammation, promoting metaplastic changes that result in the formation of osseocartilaginous nodules within the tracheal wall. Further research is needed to clarify the role of infection in TPO pathogenesis. Reported co-occurrences with TPO include malignancies such as lung cancer [15,16], retrosternal goiter [5], and, more rarely, squamous cell carcinoma of the scalp [17]. One case even described TPO mimicking recurrent papillary thyroid cancer [18]. Additionally, links to IgA deficiency have been suggested, likely due to the frequency of recurrent respiratory infections seen in affected patients [2,19]. To our knowledge, no previous cases have reported an association between TPO and hard palate perforation. While the coexistence of TPO with unrelated conditions, such as thyroid cancer and hard palate perforation in this patient, may be incidental, it highlights the need for thorough evaluation when airway abnormalities are encountered.

A Chinese cohort study of 22 patients proposed a staging classification system for TPO based on bronchoscopic and histopathological findings [20], as can be seen in Table 1. This classification divides TPO into three stages: Stage I (mild grade), Stage II (moderate grade), and Stage III (severe grade). As per this classification, the patient's findings most likely classify him into Stage 2, based on bronchoscopy revealing multiple exophytic papillomatous lesions along the anterior and lateral tracheal walls. However, definitive staging would benefit from detailed histopathological correlation. 

**Table 1:** TPO stage classification system based on bronchoscopic and histopathological findings TPO: tracheobronchopathia osteochondroplastica

Stage	Bronchoscopy findings	Histopathology findings
Stage I (Mild)	Scattered plaque-like yellow-white soft lesions on the mucosa	Inflammatory cells and occasional cartilaginous cells beneath the intact mucosa
Stage II (Moderate)	Cartilaginous nodules and sessile spicules projecting into the lumen; “cobblestone” or “stalactite cave” appearance	Nodular cartilaginous tissue
Stage III (Severe)	Deformed, rigid tracheal wall causing airway narrowing or obstruction	Prominent lamellated bone formation with fatty marrow and hematopoiesis

TPO in the patient was incidentally discovered during a hemithyroidectomy for papillary thyroid carcinoma. The patient’s history of smoking, chronic airway infection, and coexisting malignancy may have played a role in the development of the tracheal lesions. This case reinforces the importance of thorough airway assessment during unrelated procedures, especially when risk factors are present.

## Conclusions

This case illustrates the incidental detection of TPO in a patient undergoing surgery for papillary thyroid cancer. It underscores the importance of considering TPO in patients with unexplained tracheal abnormalities and highlights the role of bronchoscopy in its diagnosis. The concurrent bacterial infections in this patient further suggest a possible link between chronic infection and TPO pathogenesis. Greater awareness among clinicians is needed, especially in patients with risk factors such as smoking or recurrent airway infections. Future research should focus on clarifying the etiology of TPO and exploring treatment strategies for symptomatic cases.
